# Non-sugar sweeteners in food and beverages before the implementation of front-of-package nutrition labelling in Brazil

**DOI:** 10.1017/S1368980025101468

**Published:** 2025-12-04

**Authors:** Luiza Andrade Tomaz, Crislei Gonçalves Pereira, Sarah Morais Senna Prates, Alessandro Rangel Carolino Sales Silva, Flávia Beatriz Custódio, Lucilene Rezende Anastácio

**Affiliations:** Postgraduate Program in Food Science, Faculty of Pharmacy, Universidade Federal de Minas Gerais, Belo Horizonte, Minas Gerais, Brazil

**Keywords:** Non-sugar sweeteners, Food labelling, Front-of-package nutrition labelling, Nutritional claims, Packaged foods

## Abstract

**Objective:**

This study aimed to assess the frequency of non-sugar sweeteners (NSS) in Brazilian food products and beverages before the implementation of new nutritional labelling legislation. Specifically, we aimed to determine the eligibility of these products to contain NSS according to RDC no. 18/2008, which governed the use of NSS in Brazil during the study period.

**Design:**

Data were collected from 3335 packaged foods and beverages available in one of Brazil’s top ten supermarket chains, six months following the publication of front-of-package nutrition labelling (FoPNL) and 19 months before the legislation came into force.

**Setting:**

The study was conducted in the city of Belo Horizonte, state of Minas Gerais, Brazil.

**Results:**

Our analysis revealed that NSS were present in 12·5 % of the sampled products. Notably, high frequencies of NSS were observed in powder dessert mixes and soya drinks (100 %), gelatin preparations (88·1 %), chewing gum (87·1 %), tea (84·6 %) and carbonated beverages (71·4 %). Furthermore, we found that 82 % of products containing NSS made claims regarding sugar and calorie reduction, with 16·6 % of these claims being inconspicuous. Additionally, 14 % of products targeted controlled sugar intake diets, 0·5 % aimed at sugar-restricted diets and 4 % were ineligible for NSS use. Importantly, the declared NSS content adhered to Brazilian regulatory limits in beverages.

**Conclusions:**

While most products complied with regulatory standards, our findings highlight the presence of ineligible products and less prominent claims, which may complicate NSS identification for consumers. Continuous monitoring of NSS frequency, especially following the implementation of FoPNL, is essential for ensuring compliance with regulations and promoting informed consumer choices in Brazil.

Non-sugar sweeteners (NSS) are substances other than sugars that impart a sweet taste to food and beverages^(1)^. Their use allows sweetness without a significant increase in caloric content^(2)^. In Brazil, additives must be used in limited food products, under specific conditions, and at the lowest level to achieve the desired effect. Brazilian regulations establish that the use of NSS is allowed only in foods and beverages with partial or total sugar replacement^(3)^. These products include foods and beverages for weight control, diets with restricted or controlled sugar intake, or those with claims such as ‘low’ or ‘reduced’ sugars or energy value, or ‘does not contain’ or ‘without added sugars’^(3)^.

The consumption of free sugars has been linked to increased obesity and chronic non-communicable diseases^(4)^. The WHO recommends limiting daily caloric intake from free sugars to 10 %, equivalent to 50 g of added sugar in a 2000-calorie diet^(5)^. As indicated in the Scientific Report of the 2015 Dietary Guidelines Advisory, reducing sugar intake in the diet should primarily involve adopting healthier habits, such as replacing sugary beverages with water, rather than substituting sugars with NSS^(6,7)^.

Although the safety conditions of using food additives were internationally evaluated by the Joint FAO/WHO Expert Committee on Food Additives (JECFA)^(8)^, acceptable daily intake (ADI) was defined for each NSS separately^(8)^, without considering synergistic studies with associated intake of them, as evidence of these synergistic effects on health is still scarce. However, the population is often exposed to multiple NSS by foods and beverages^(9)^.

Some adverse effects were already recognised for establishing the ADI of NSS, such as maternal toxicity in a developmental toxicity study for advantame; caecal enlargement for acesulfame potassium; reproductive effects for aspartame, cyclamate and steviol glycosides; effects on kidneys for cyclamate; increase in alkaline phosphatase activity for neotame; body weight changes for sucralose; and disturbance of homoeostasis for saccharin^(8)^. However, the current literature on the consequences of NSS consumption is divergent. Some recent studies have demonstrated the negative effects of some sweeteners on the alteration of microbiota and development and worsening of glucose intolerance^(10,11)^, even at doses below the ADI^(11)^, and transfer of sweeteners from breast milk to the circulation of infants^(12)^. A comprehensive systematic review and meta-analysis conducted by the WHO, encompassing 283 studies, yielded disparate findings regarding the impact of NSS on conditions such as adiposity and type 2 diabetes^(4)^. In this sense, WHO guidelines discourage using NSS to control body weight or reduce the risk of non-communicable diseases^(4)^.

Despite this uncertainty, the global utilisation of NSS has increased^(13–15)^. Per capita volumes of NNS from beverage sales increased by 36 % globally, with a significant increase in regions with more policy actions to reduce the consumption of added sugars^(16)^. Some authors attribute this rise to regulatory measures, including the implementation of front-of-package nutrition labelling (FoPNL)^(9,17)^.

In Chile, following the enactment of Law 20606/2012, which included FoPNL, there was a notable rise in the prevalence of products containing NSS^(9,18)^. Anticipating adverse effects, Mexico, Argentina and Colombia introduced regulations mandating the inclusion of NSS information on the front label of products, aiming to discourage their consumption by children^(19–21)^. There has been widespread advocacy for clearer disclosure of these additives on food labels to facilitate more informed consumer decisions^(18,22–24)^.

In Brazil, a reviewed legislation regarding nutrition labelling (RDC no.429/2020^(25)^ and IN no.75/2020^(26)^) was approved in October 2020, becoming effective in October 2022. Under this legislation, products with high levels of added sugar (> 15·0 g/100 g or >7·5 g/100 ml), saturated fat (> 6·0 g/100 g or > 3·0 g/100 ml) and Na (> 600 mg/100 g or > 300 mg/100 ml) will receive the FoPNL ‘high in’ with a magnifying glass design, in the upper half of the front panel^(25,26)^, as shown in Figure 1.

**Figure 1.**
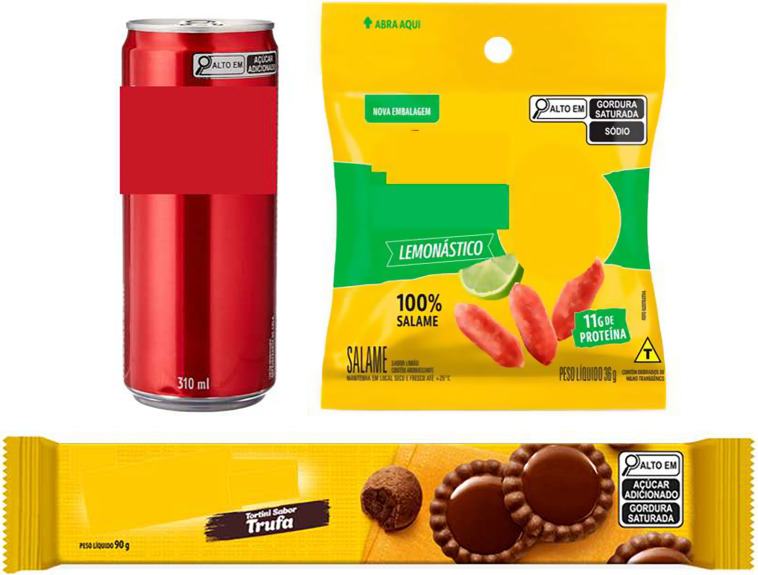
Examples of labels with the Brazilian front-of-package nutrition labeling (high in magnifying glass design) of food products sold in Brazil.

Studies on the frequency of NSS in Brazilian products have been conducted^(23,27,28)^, but there is no characterisation of products with NSS based on Brazilian legislation^(3)^. Due to this scenario, it is not known whether NSS have been used only in food and beverage with partial or total sugar replacement and in levels in accordance with current legislation. Another gap identified is whether the frequency of NSS usage may be affected by the new Brazilian labelling legislation, especially by the FoPNL. In this way, this work aimed to evaluate the frequency of NSS in Brazilian products before the implementation of new nutritional labelling legislation, verifying their eligibility to receive this additive according to RDC no. 18/2008^(3)^, and whether the declared contents were within established limits.

## Methods

### Type of study and data collection

This is a cross-sectional, descriptive, quantitative study conducted by surveying the labelling of food and beverages in a supermarket chain in the city of Belo Horizonte, MG. Belo Horizonte is the sixth most populous city in Brazil, the capital of the state of Minas Gerais in the southeast region, with 2 315 560 inhabitants in 2022^(29)^.

Data collection occurred from March to May 2021 and was carried out by trained collectors who had obtained prior authorisation from the establishment’s management. The supermarket was selected for convenience and considering it is in the eighth position among the twenty largest supermarket chains in Brazil for 2020, according to the ranking published by the Brazilian Supermarket Association (ABRAS)^(30)^.

Information from all products featuring a nutritional information table available for sale during the data collection period was gathered using the Epicollect 5 software^(31)^. This is a free data collection application that can be used on both mobile devices and web browsers^(31)^. All variations of products, including different sizes or flavours, were included to represent the total number of products available for sale in the supermarket. The same products (same brand and sales denomination) in more than one package size represented 14 % of the database (*n* 467).

The collected data comprised the commercial name, sales denomination, flavour, liquid content, brand, barcode and images of the front panel, nutritional information table and list of ingredients. Following collection, the data were transferred to a Microsoft® Excel spreadsheet. In this spreadsheet, details from the nutritional information table, label claims and information regarding NSS, such as their presence, type, number and content (for beverages), were recorded. The products were classified according to IN no. 75/2020^(26)^, with adaptations. Additional details about the data collection procedures can be found in Tomaz *et al.* ^(32)^.

### Evaluation of the presence of non-sugar sweeteners

To determine the presence of NSS, RDC no. 18/2008^(3)^, which regulated the use of NSS in Brazil during the study period, was consulted. Subsequently, the lists of ingredients of all foods in the database were examined, scanning for terms established in RDC no. 18/2008^(3)^, such as sorbitol, sorbitol syrup, D-sorbite, mannitol, isomalt, isomaltitol, maltitol, maltitol syrup, lactitol, xylitol, erythritol, acesulfame potassium, aspartame, cyclamic acid and its Ca, potassium, and Na salts, saccharin and its Ca, potassium, and Na salts, sucralose, neotame, advantame, thaumatin and steviol glycosides. Variations of these names, like ‘stevia’, were also taken into account, as carried out in the study by Figueiredo *et al.* ^(28)^, since some products declared the presence of NSS without the standardisation established in the legislation. Polyols can be used with different functions, but they were considered only when it was declared they were used with the NSS function. The types and quantity of NSS identified per product were then outlined in both relative and absolute frequencies.

According to Brazilian legislation, food additives are required to be disclosed in the list of ingredients, specifying the primary or essential role of the additive in the food, accompanied by its complete name or International Numbering System (INS) code, or both^(33)^. Accordingly, products were categorised as ‘containing NSS’ only when the aforementioned food additives were preceded by a declared sweetening function.

### Eligibility of products with non-sugar sweeteners

Foods and beverages fitting within authorised categories by RDC no. 18/2008^(3)^ were deemed eligible for NSS use^(3)^. To comprehend the labelling of products for weight control and diets with restricted or controlled sugar intake, Ordinances No. 29^(34)^ and 30^(35)^ of 1998 were consulted (legislation applicable during the study period). As per these regulations, product labels must specify sales denominations, presenting the name of the product followed by its purpose^(34,35)^.

For eligible products with claims about partial/total reduction of sugars and/or caloric content, RDC no. 54/2012^(36)^ (in force during the study period) was referenced to verify the authorised terms/synonyms for the attributes ‘reduced’, ‘low’, ‘very low’, ‘does not contain’ and ‘without added’. The presence of nutritional claims was evaluated individually – label by label, using photos of the products, recording the respective term used and where on the label the claim was located, whether on the front, side or back panel.

### Evaluation of declared non-sugar sweetener contents

The NSS contents declared on labels were verified and compared with the limits established by Brazilian legislation^(3)^. In Brazil, only diet drinks and low-calorie drinks are required to declare NSS content in the list of ingredients^(37)^. According to Decree no. 6871/2009^(37)^, these beverages are classified as non-alcoholic and low-calorie, with their sugar content completely substituted by low-calorie or non-caloric sweeteners, whether natural or artificial, either individually or in combination^(37)^. The labelling of these beverages is required to indicate the generic name of the NSS, their category and the quantity in weight per unit or milligrams per hundred millilitres.

To assess NSS contents, maximum levels were consulted in RDC no. 18/2008^(3)^ for categories including foods and beverages for weight control, diets with controlled sugar intake, sugar-restricted diets and those with claims involving total or partial sugar replacement. Thus, each beverage declaring NSS content was evaluated within its respective category. For products ineligible for NSS use, the list of ingredients was examined. If both sugar and NSS were present, the defined limits for foods and beverages with claims involving partial sugar replacement were considered. If products lacking NSS eligibility contained only NSS, the defined limits for foods and beverages with claims involving total sugar replacement were considered^(3)^.

### Statistical analysis

Microsoft® Excel version 2013 was employed for data tabulation and analysis. Descriptive statistical analysis, presenting categorical data with percentages and absolute numbers, was conducted.

## Results

### Profile of non-sugar sweetener use in foods and beverages

**Figure 2.**
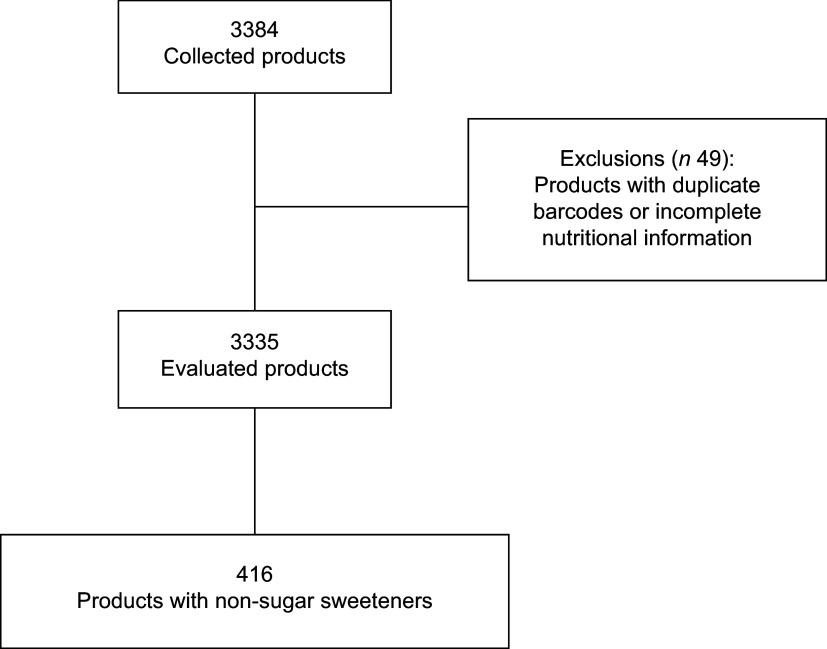
Sample flow chart.

Data from 3384 products were collected, with forty-nine products excluded due to duplicate barcodes or incomplete nutritional information. This exclusion resulted in a sample of 3335 products (Figure 2). Among these, 416 (12·5 %) contained at least one NSS, spread across thirty-eight food and beverage categories. Notably, 100 % of products in the categories of powders for preparing flans and desserts (*n* 13) and soya-based beverages (*n* 5) included NSS. Additionally, other categories exhibited high frequencies of products with NSS, including powders for gelatins (*n* 52; 88·1 %), chewing gum (*n* 7; 87·5 %), teas (*n* 11; 84·6 %) and soft drinks (*n* 45; 71·4 %) (Table 1).

**Table 1.** Presence of NSS by type of product, in absolute and relative frequency in categories that had at least one product with NSS

Food and beverages	Absolute and relative frequency of products with NSS	
	*N*	%
Cakes, breakfast cereals and baked goods	24	10·5 %
Cakes, all types without filling	4	28·6 %
Cakes and similar with filling or frosting	2	8·0 %
Powders for preparing cakes and pies	4	9·3 %
Breakfast cereals weighing up to 45 g per cup	6	16·2 %
Sliced or unsliced packaged breads, with or without filling	5	6·5 %
Sweet breads without fruit	1	11·1 %
Potato bread, cheese bread and other chilled and frozen breads without filling	2	8·3 %
Non-alcoholic drinks	143	38·5 %
Soya-based drinks	5	100 %
Fruit drinks	8	25·0 %
Nectars	24	46·2 %
Teas	11	84·6 %
Soft drinks	45	71·4 %
Electrolyte drinks	3	23·1 %
Powders for refreshment preparation	45	25·7 %
Other drinks	2	9·5 %
Dairy products	59	19·5 %
Dairy drinks	12	16·2 %
Yogurts	39	23·6 %
Fermented milks	5	15·6 %
Petit-suisse cheese	3	9·4 %
Products with energy mainly from sugars and/or fats	180	20·8 %
Powdered chocolate mix, cocoa-based powders, chocolate powder and cocoa powder	9	18·8 %
Candies, lollipops and pastilles	3	5·3 %
Cereal bars with more than 10 % of fat, nougat, *pé de moleque* and *paçoca*	3	12·5 %
Sweet biscuits, with or without filling	36	12·6 %
Chocolates, chocolate candies and similar	15	9·9 %
Solid sweets (guava, quince, fig, potato, etc)	3	10·7 %
Pasted sweets (pumpkin, guava, milk, banana, *mocotó*)	8	36·4 %
Jams	13	30·2 %
Chewing gum	7	87·5 %
Powders for preparing gelatin	52	88·1 %
Powders for preparing flans and desserts	13	100 %
Ice creams	7	7·1 %
Fruit in syrup	2	50 %
Dairy desserts	4	36·4 %
Peanut butter	5	41·7 %
Sauces	10	18·5 %
Tomato sauce	5	20 %
Barbecue sauce	1	33·3 %
Ketchup and mustard	4	15·4 %
Total	416	12·5 %

NSS, non-sugar sweeteners.

**Figure 3.**
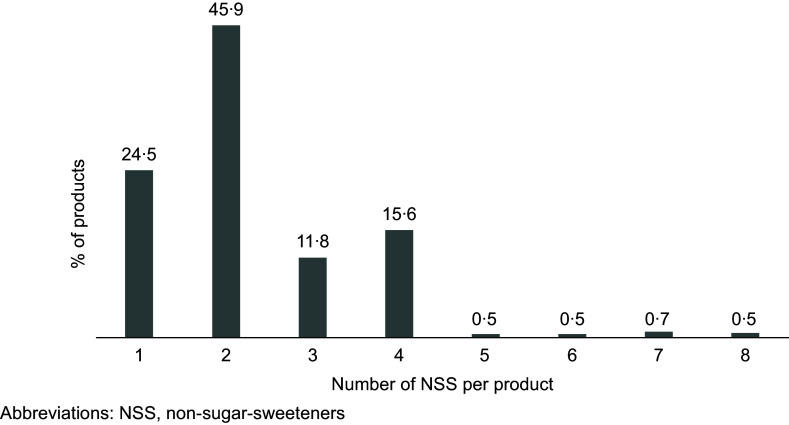
Relative frequency of the quantity of declared NSS per product. NSS, non-sugar sweeteners.

Regarding the number of NSS, the majority of products (75·5 %) had more than one NSS (Figure 3). NSS were mentioned 939 times, with sucralose being the most cited, found in 198 products (47·6 %), followed by acesulfame potassium in 183 products (44 %), and cyclamate and its salts in 129 products (31 %). Advantame did not appear in any product (Table 2). Steviol glycosides and sucralose were the only NSS declared alone on the evaluated labels. Steviol glycosides were the sole NSS in cakes, nectars, dairy drinks, fermented milk, petit-suisse cheese and teas. Sucralose was the sole NSS in breads, tomato sauce, dairy drinks, peanut butter, solid sweets, ketchup, mustard, cola and electrolyte drinks. The other NSS were consistently combined, with the primary combination being acesulfame K and sucralose (*n* 57, 13·7 %).

**Table 2.** Relative and absolute frequency of NSS in 416 products

NSS	Absolute frequency	Relative frequency (%)
Sucralose	198	47·6
Acesulfame potassium	183	44·0
Cyclamate	129	31·0
Aspartame	113	27,2
Saccharin	108	26·0
Steviol glycosides	70	16·8
Maltitol	56	13·5
Sorbitol	46	11·1
Xylitol	13	3·1
Thaumatin	8	1·9
Erythritol	7	1·7
Isomalt	3	0·7
Mannitol	2	0·5
Neotame	2	0·5
Lactitol	1	0·2
Advantame	0	0·0

NSS, non-sugar sweeteners.

The presence of sorbitol and maltitol polyols without a declared sweetening function was observed in sixty-two products (1·8 %). Among these, seventeen (27·4 %) had concomitant additives with a declared sweetening function, while the remaining forty-five (72·6 %) did not. Products exclusively containing polyols with another declared technological function or without a specified function belonged to various categories: cakes and similar products with filling or frosting (*n* 17), all types of cakes without filling (*n* 10), cereal bars with up to 10 % of fat (*n* 3), cereal bars with more than 10 % of fat, nougat, pé de moleque, and paçoca (*n* 3), sweet cookies with or without filling (*n* 4), candies, lollipops, and pastilles (*n* 4), chocolates, chocolate candy, and similar (*n* 2), brownies and alfajores (*n* 1), and whipped cream (*n* 1). These products were not considered as products with NSS, since Brazilian law establishes that food additives must be included in the list of ingredients preceded by their primary or essential function. The functions and the absolute frequency of citations of polyols with a declared non-sweetening function are indicated in Table 3.

**Table 3.** Absolute frequency of citations of polyols with a function declared that is non-sweetening in sixty-two products

Function	Sorbitol	Maltitol
Humectant	35	0
Stabiliser	7	12
Emulsifier	4	0
Mass agent	1	0
Not specified	7	7
Total	54	19

### Eligibility of products to use non-sugar sweeteners

Regarding the eligibility of the 416 products with NSS, 401 products (96 %) were authorised to use these additives according to RDC no. 18/2008. The fifteen products (4 %) that contained NSS in their composition, but were not eligible for use, consisted of yogurt (*n* 4), ketchup (*n* 2), peanut butter (*n* 2), dairy drink (*n* 1), cappuccino (*n* 1), curd (*n* 1), soft drink (*n* 1), tomato sauce (*n* 1) and bread (*n* 1).

**Figure 4.**
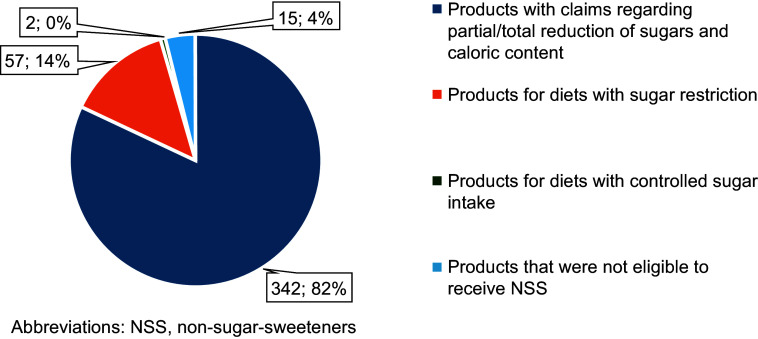
Profile of the 416 foods with NSS, in absolute and relative frequency. NSS, non-sugar sweeteners.

Of the 416 products, 342 (82 %) were eligible for claims related to attributes like ‘low’ or ‘reduced’ in sugars or energy value, or ‘do not contain’ or ‘no added sugars’ (Figure 4). The most common claim in products with NSS was ‘without added sugars’, present in 144 products, while the less prevalent claim was ‘low in sugars’, found in eighteen products (Table 4). Notably, all foods for diets with controlled sugar intake (*n* 57; 100 %) and diets with sugar restriction (*n* 2; 100 %) also had some type of claim. The term ‘diet’, optionally used for weight control foods, diets with controlled intake of sugars and diets with restricted nutrients, appeared in twenty-seven products, with twenty-three being foods for diets with controlled intake of sugars and four having a claim.

**Table 4.** Frequency of claims regarding partial/total reduction of sugars and caloric content in products with NSS, in absolute frequency

Claim	Foods with claims	Foods for D.C.S.I.*	Foods for D.S.R.**	Total occurrence of claims
Claim (does not contain sugar)	87	15	1	103
Sugar-free	0	0	0	0
Zero (0 or 0 %) sugar	62	11	1	74
No sugar	25	4	0	29
Without sugar	0	0	0	0
Claim (no added sugars)	107	35	2	144
Without addition of sugar	41	17	1	59
Zero added sugar	64	18	1	83
No added sugar	2	0	0	2
Claim (low in sugars)	18	0	0	18
Low in sugar	18	0	0	18
Little sugar	0	0	0	0
Low sugar content	0	0	0	0
Light on sugar	0	0	0	0
Claim (reduced in sugar)	60	0	0	60
Reduced in sugar	41	0	0	41
Less sugar	18	0	0	18
Lower sugar content	0	0	0	0
Light	1	0	0	1
Claim (low in energy value)	53	0	0	53
Low in energy value	32	0	0	32
Low in calories, kcal or kilocalories	18	0	0	18
Little energy value	0	0	0	0
Little calories, kcal or kilocalories	0	0	0	0
Low content of…	0	0	0	0
Light on calories	3	0	0	3
Claim (reduced in energy value)	25	2	1	28
Reduced in energy value	6	0	0	6
Reduced in calories, kcal or kilocalories	1	0	0	1
Fewer calories, kcal or kilocalories	10	2	1	13
Less calories, kcal or kilocalories	0	0	0	0
Light	8	0	0	8

NSS, non-sugar sweeteners; *D.C.S.I., diets with controlled sugar intakes; **D.S.R., diets with sugar restriction.

**Figure 5.**
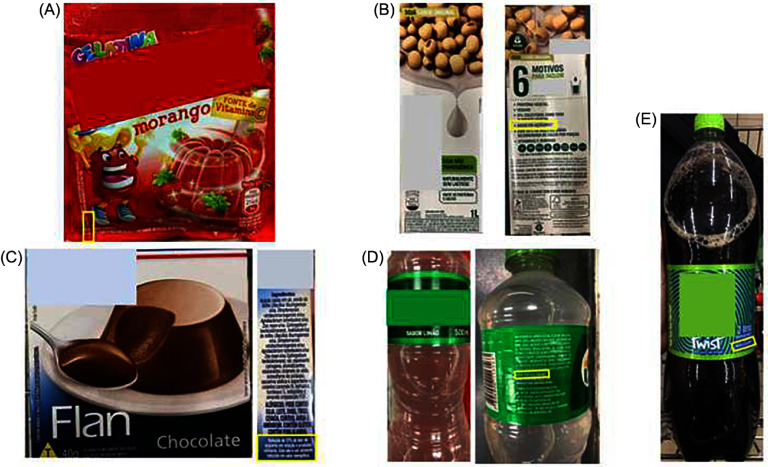
Examples of products with NSS with claims referring to sugar and/or calories in a less prominent place. (A) “Low in calories” in small print on the lower left corner of the front panel of the label; (B) “low in sugars” on the side panel of the label; (C) “97% reduction in sugar content compared to similar products” on the side of the label; (D) “light in calories” on the back of the label, after the ingredients list; (E) “low calorie” next to the sales denomination. NSS, non-sugar sweeteners.

It is worth mentioning that among the 342 eligible for claims, fifty-seven products (16·6 %) presented this information on the side or back panel of the label, with forty-five (13·2 %) indicating the claim after the list of ingredients. Additionally, in the non-alcoholic beverage category, ten products (3 %) only had the claim next to the sales name. Examples are displayed in Figure 5.

### Declared non-sugar sweetener content

Out of 416 products with NSS, 115 (27·6 %) declared their content. Among these, fifty-one (44·3 %) were diet and low-calorie beverages, and sixty-four (55·7 %) were low or reduced-sugar beverages. Notably, another twenty-seven (6·5 %) low-calorie beverages did not declare the NSS content, despite it being mandatory. In terms of NSS-level evaluation, all assessed products were within the limits established by RDC no. 18/2008.

Among the beverages that declared NSS contents (*n* 115), acesulfame K was the most cited (*n* 92), followed by sucralose (*n* 48). The proportions of NSS content in relation to the maximum permitted limit (MPL) are presented in Table 5. The declared amounts of NSS ranged, on average, from 23 % to 38 % of the respective MPL. Sodium cyclamate content in some products was close to 100 % of the MPL.

**Table 5.** Declared NSS contents in beverages

NSS	Minimum content (mg/100ml)	% in relation to MPL – RDC 18/2008	Maximum content (mg/100ml)	% in relation to MPL – RDC 18/2008	Average content (mg/100ml)	Codex maximum limits (mg/100ml)
Acesulfame K	1·6	4·7	29·0	82·8	8·0	11–500
Aspartame	6·6	11·8	45·0	60·0	25·6	4–30
Sodium cyclamate	2·0	2·7	70·0	93·3	24·6	3–1000
Steviol glycosides	11·0	18·3	19·0	42·2	17·3	25–300
Sodium saccharin	1·0	10·0	13·3	88·7	4·2	8–250
Sucralose	1·6	8·0	18·0	72·0	7·3	1–100

## Discussion

### Profile of non-sugar sweetener use in foods and beverages

The frequency of NSS use found in this study was 12·5 %. Notably, the data were collected six months after publication and a year and a half before the new regulation on Brazilian nutrition labelling came into force. In previous national studies, such as Figueiredo *et al.* ^(28)^, who evaluated 4539 products collected in 2013, the frequency of NSS found was 13·3 %. Another study by Grilo *et al.* ^(23)^, evaluating 11 434 products collected in 2017, reported a frequency of NSS use of 9·3 %.

Studies on the use of NSS in food and beverages have been conducted worldwide, with different observed percentages, such as 4·5 % in Hong Kong^(38)^, 8·8 % in Turkey^(15)^, 9·3 % in Spain^(24)^, 16 % in Colombia^(39)^ and 55·5 % in Chile^(9)^. It is important to highlight that these differences can be attributed to the selection of products evaluated, such as, for example, the study conducted in Hong Kong^(38)^ that focused exclusively on non-diet products, and to the nutritional profile of the products, which may vary according to the local context. The high percentage of sweeteners observed in Chile^(9)^ might be due to the evaluation of products that are expected to use sugar in their composition and their reformulation in response to the implementation of FoPNL.

Considering the frequency of NSS per food and beverage category, there was a likely increase in the use of this additive in the categories of powders for preparing flans and desserts and soft drinks compared to previous studies in Brazil. In the work by Figueiredo *et al.* ^(28)^, the frequency of NSS in the category of powders for preparing flans and desserts was 58·3 % (*n* 14), and in the work by Grilo *et al.* ^(23)^, the presence of NSS in soft drinks was 44·3 % (*n* 47). In the present study, the frequency was 100 % (*n* 13) in powders for preparing flans and desserts and 70·2 % (*n* 40) in soft drinks. Since data collection for this study took place six months after the publication of the legislation implementing FoPNL in Brazil, it is possible that this increase was a consequence of the industry reformulating products to avoid FoPNL for sugar, as it has already been observed in Chile^(9)^. Another possibility is the replacement of sugar for NSS to make the production chain of these products cheaper, since the sweetening power of NSS is greater, resulting in lower expenses not only in the composition of the products but also in their transportation, making shipping cheaper.

A study by Hafner and Pravst^(14)^ investigated recent changes in the use of NSS in more than 1000 non-alcoholic beverages marketed in Slovenia. The work indicated that the frequency of NSS use in beverages in the years 2015, 2017 and 2020 was 13·2 %, 15·5 % and 20·2 %, respectively. The presence of NSS in soft drinks in the same years was 16·8 %, 19·6 % and 26·7 %, respectively. These findings indicated an increase in the frequency of NSS use, as well as the reformulation of beverages. The frequency of NSS in soft drinks indicated in this study was higher than that found in Slovenia and similar to Spain, where 78·1 % of soft drinks had NSS^(24)^. Carvalho *et al.* ^(40)^ indicated that soft drinks were the main source of exposure for most NSS in the Portuguese population.

Some evaluated categories in this work had 100 % of the products with NSS. The shortage of products without NSS in certain food categories reduces consumer choice, as depicted in the literature. A study by Sambra *et al.* ^(9)^ with 1489 Chilean food products and beverages containing sugars and/or NSS revealed that more than 90 % of foods in the categories of powdered juices, jellies, waters and flavoured milks contained NSS. In the work by Beltrá *et al.* ^(24)^ with Spanish food products, it was found that some brands did not have any drinks without NSS, while others only had a few versions free of these additives.

As observed in this study, the increase in sweetener use appears to be driven primarily by beverages, a finding also reported in other studies^(9,41)^. This growth reflects a response to the growing interest in reducing the consumption of free sugars in the diet^(41)^, although the recommendation of international authorities is that reducing free sugar levels should not result in the addition or replacement of sweeteners^(7)^. Survey by Goodman *et al.* ^(42)^, for example, found that a total of 65·5 % consumers reported efforts to reduce their sugar consumption, compared to only 31·2 and 24·0 % who tried to decrease their intake of low-calorie artificial sweeteners and natural sweeteners, respectively. These choices appear to be more associated with the perceived ‘naturalness’ of sweeteners than with their energy content^(42)^.

Although NSS is a low- or no-calorie alternative to free sugars^(4)^, this wide substitution raises complex questions regarding their long-term health implications, since this can increase the dietary exposure of the Brazilian population to NSS and this exceeds the toxicological references, that is, ADI, or also it can lead to other effects considering there is still no clear consensus on some NSS effects^(4)^. The WHO, for example, does not recommend using NSS for weight control or reducing the risk of non-communicable diseases^(4)^. Furthermore, the high prevalence of sweeteners in processed foods and beverages limits consumer choice and is of particular concern for the paediatric population^(9)^.

Regarding the most used NSS, sucralose and acesulfame potassium were the most frequent in this study, consistent with the findings of Takehara *et al.* ^(43)^ and Grilo *et al.* ^(23)^. A similar trend was observed in the study by Figueiredo *et al.* ^(28)^, where acesulfame potassium led, followed by sucralose. This higher frequency of sucralose and acesulfame potassium observed in the present study is likely due to their technological characteristics. About sucralose, specifically, it is expected that its higher frequency is due to its lower aftertaste and sweetness closer to sucrose, in addition to the fact that it can be used alone and not in combination with another sweetener. Regarding acesulfame potassium, its physical-chemical stability allows it to be used in products that are submitted to higher temperatures such as baked products^(44)^. In addition, its long shelf life allows it to be widely used in various types of products^(45)^.

Noteworthy changes in the types of NSS used in the Brazilian scenario were observed. In the study by Figueiredo *et al.* ^(28)^, no evaluated product had erythritol or thaumatin, while in the present study, erythritol was cited seven times, and thaumatin was cited eight times. Grilo *et al.* ^(23)^ reported the presence of erythritol in ten products and thaumatin in twelve products. It is worth noting that advantame, approved for use in Brazil in 2019^(46)^, did not appear in any of the evaluated products in this study, but Takehara *et al.* ^(43)^ cited it twice in 1869 products with NSS. This low frequency of use of advantame in Brazil is likely due to its relatively recent approval^(46)^.

In this study, when the product only had polyols with another declared technological function or without a specific function, it was not considered a product with NSS. This decision was based on the stated purpose on the label, which was not for sweetening, making it challenging to evaluate it considering RDC no. 18/2008^(3)^. Polyols often serve purposes like providing bulk or texture rather than sweetness^(47)^. Sorbitol and maltitol polyols appeared in the evaluated products in this work with functions like humectant, stabiliser, emulsifier, bulking agent and sweetening. In Brazil, seven functions are authorised for sorbitol, including body or mass agent, NSS, thickener, stabiliser, sequestrant and humectant, while maltitol has four authorised functions: body or mass agent, NSS and stabiliser^(48)^.

The use of NSS in food and beverages in Brazil is regulated by RDC no.18/2008^(3)^ and is permitted only in specific situations, acting as a measure to control the indiscriminate use of the additive. This study revealed that 4 % (*n* 15) of the evaluated products with NSS did not provide justification for the use of this additive. Concerning NSS content, the beverages that declared the content were within the established limits. The proportions of NSS use in the evaluated beverages ranged from 2·7 % to 93·3 % of the maximum limit established by Brazilian legislation, with sodium cyclamate being closest to 100·0 %. Data on NSS content in Brazilian food and beverages are still scarce in the literature. The NSS levels found in sixty-four samples of non-alcoholic beverages in Brazil^(49)^ were 54·1 up to 194·0 % of declared levels and 11 % of samples with declared NSS on label were not within the Brazilian MPL. These maximum limits aim to ensure that the intake of an additive does not exceed the ADI^(50)^ and monitoring NSS in food is still an important necessity.

Furthermore, it is important to emphasise that there is still no clear consensus on the effects of ingesting sweeteners even at doses within the ADI^(4)^. A study by Suez *et al.* ^(10)^, for example, indicated that non-caloric artificial sweeteners may favour the development of glucose intolerance by inducing changes in the composition and functionality of the intestinal microbiota. Subsequently, another study conducted by the same authors^(11)^ observed a worsening of glucose tolerance with the ingestion of saccharin and sucralose, even at consumption levels within the ADI, of 20 % and 34 % of the daily recommendation, respectively. Therefore, studies that estimate the average intake of sweeteners by the population are essential to better understand the intake patterns and possible associated risks.

Considering RDC no.588/2021^(51)^, which lowered the maximum limit of NSS steviol glycosides allowed for use in foods and beverages, four drinks in this study would have exceeded the updated limits. Nevertheless, as this legislation was published in December 2021^(51)^ and came into force in January 2022, after data collection for this work, the limits previously established by RDC no.18/2008^(3)^ for NSS steviol glycosides were considered.

The combination of two or more NSS occurred in 75·5 % of the products with declared NSS evaluated in this study. In Chile, this combination was found in 63·9 %^(9)^ of the evaluated products, and in Spain, it was 65·7 %^(24)^. This practice can prevent undesirable residual flavours, enhance the sweetness of the components and minimise the chances of exceeding the ADI^(52)^. However, the interaction of these substances and their consequences remains unclear^(4,9)^.

### Eligibility of products for the use of non-sugar sweeteners

Most of the evaluated products with NSS in this study were eligible to receive the additive, as they had a claim referring to sugar or calories (*n* 342; 82 %). A study by Beltrá *et al.* ^(24)^, with 4218 products marketed in Spain, indicated that of the 301 evaluated products with NSS, 94·1 % had some nutritional or health claim, and of these, 44·5 % were nutritional claims referring to sugar or energy. The study by Grilo *et al.* ^(23)^, in which 3491 Brazilian products were evaluated, revealed that 56·8 % of products with NSS had some claim related to NSS.

It should be noted that in 16·6 % (*n* 57) of the eligible products for receiving NSS, as they had a claim referring to sugar or calories, the claim was in a place of little prominence. Nutritional claims are used as a marketing strategy and can be found on the front (main), back and side of the package^(53–55)^. For the packages, we must consider all their dimensions, regardless of the format, since the buyer can analyse the entire area of the product^(56)^. However, the front panel is where the most important product information is concentrated, and this is the most obvious location for the consumer^(55)^.

The presence of the claim referring to the reduction of sugar or calories, in a place of low visibility, seems to indicate that the use of these claims is more linked to compliance with Brazilian legislation^(3)^, giving the product eligibility to have an NSS, than to be a marketing strategy or offer of a product with particular nutritional properties. The use of claims aims to facilitate consumer knowledge about the nutritional properties of foods, contributing to its proper selection^(36)^. However, often when choosing products with a claim referring to sugar or calories, the consumer does not imagine that these products may have NSS^(22)^. And this can be even worse when the claim is not easily perceived, which can lead the consumer to involuntary consumption of the additive. It is important to mention that low-sugar foods, which have NSS and added sugar, may result in unintentional NSS intake, since the consumer may assume that sugar-sweetened foods do not contain NSS^(21)^. The increase in involuntary consumption of NSS has already been reported in the literature. A study by Sylvetsky *et al.* ^(57)^ indicated the presence of sucralose in the urine of 44 % of the participants in the USA, who reported no consumption of these additives.

### Declared non-sugar sweetener content

The declaration of NSS content on labels is not among the recommendations of the Codex Alimentarius. However, the non-declaration of NSS content in food hinders studies with accurate estimates of NSS consumption by the population. In Brazil, the declaration of NSS content is only mandatory on the labels of diet and low-calorie drinks^(37)^, which limits the assessment of the content of these additives in all products.

Attempts to obtain the declaration of content in other products are unprofitable, as mentioned in the work by Carvalho *et al.* ^(40)^. The USA has a similar situation; the Food and Drug Administration (FDA) determines the declaration of NSS in the list of ingredients of products that contain the additive, but not the content. However, authors discuss the importance of such information being made available on labels^(22)^. In countries such as Mexico and Chile, this information is available on product labels^(58,59)^. Additionally, Mexico, Argentina and Colombia foresee the adoption of a warning about the presence of NSS on the front panel of the label^(19–21)^.

### Strengths and limitations of the study

The strengths of the present study include the evaluation of the eligibility of food products for the use of NSS according to Brazilian legislation and the utilisation of a recent database. This allows us to understand the scenario shortly before the implementation of the new legislation on nutrition labelling and may assist in monitoring the frequency of this additive in food products.

Regarding limitations, there are some points that deserve discussion. Data collection occurred in only one supermarket in Belo Horizonte, MG, representing part of the scale of packaged foods available for sale in Brazil. Additionally, the data collection did not include energy drinks. Also, the inclusion of products in their different packaging sizes may interfere in the percentage of products with NSS. Finally, the various ways of categorising a database can make it challenging to compare our results with those from different studies.

## Conclusion

The frequency of NSS in Brazilian products prior to the implementation of the new nutritional labelling legislation was 12·5 %. Categories such as powders for preparing flans and desserts and soya-based drinks were notable for the absence of options without NSS. Other categories stood out due to a high frequency of NSS, including powders for preparing gelatins, chewing gum, teas and soft drinks. In this study, the majority of evaluated products with NSS were eligible to receive the additive, with 82 % citing justifications aligned with the claims outlined in RDC no.18/2008. However, this information was sometimes located in less prominent places, potentially complicating the identification of NSS in the product and leading consumers to errors and deception during purchases. It is essential to continue monitoring the frequency of NSS in products, especially in the post-implementation scenario of Brazilian nutritional labelling standards and related health policies.

## References

[1] Ministério da Saúde & Secretaria de Vigilância Sanitária (1997) Portaria n. 540, de 27 de outubro de 1997. Technical regulations: Food Additives - definitions, classification, and use. https://bvsms.saude.gov.br/bvs/saudelegis/svs1/1997/prt0540_27_10_1997.html (accessed May 2021).

[2] Carocho M , Morales P & Ferreira ICFR (2017) Sweeteners as food additives in the XXI century: a review of what is known, and what is to come. Food Chem Toxicol 107, 302–317. 28689062 10.1016/j.fct.2017.06.046

[3] Ministério da Saúde & Agência Nacional de Vigilância Sanitária (2008) Resolução da Diretoria Colegiada - RDC n. 18, de 24 de março de 2008. Provides for the Technical Regulation that authorizes the use of sweetener additives in foods, with their respective maximum limits. https://bvsms.saude.gov.br/bvs/saudelegis/anvisa/2008/rdc0018_24_03_2008.html (accessed 03 May 2022).

[4] Rios-Leyvraz M & Montez J (2022) Health Effects of the Use of Non-Sugar Sweeteners: A Systematic Review and Meta-Analysis. https://www.who.int/publications/i/item/9789240046429 (accessed November 2024).

[5] World Health Organization (2015) Guideline: Sugars Intake for Adults and Children. Geneva: WHO. 25905159

[6] Millen BE , Abrams S , Adams-Campbell L et al. (2016) Scientific report of the 2015 dietary guidelines advisory committee. Adv Nutr 7, 438–444. 27184271 10.3945/an.116.012120 PMC4863277

[7] Ministério da Saúde & Secretaria de Atenção à Saúde (2018) Departamento de Atenção Básica. Coordenação-geral de Alimentação e Nutrição. *Sugar Reduction Plan for processed foods.*. https://www.gov.br/saude/pt-br/composicao/saps/promocao-da-saude-e-da-alimentacao-adequada-e-saudavel/reducao-de-sodio-acucar-e-gordura-trans/materiais-de-apoio/plano_reducao_acucar_alimentos.pdf/view (accessed 29 November 2021).

[8] JECFA (2022) Evaluations of the Joint FAO/WHO Expert Committee on Food Additives (JECFA). https://apps.who.int/food-additives-contaminants-jecfa-database/ (accessed November 2024).

[9] Sambra V , López-Arana S , Cáceres P et al. (2020) Overuse of non-caloric sweeteners in foods and beverages in Chile: a threat to consumers’ free choice? Front Nutr 7, 68. 32626722 10.3389/fnut.2020.00068 PMC7311776

[10] Suez J , Korem T , Zeevi D et al. (2014) Artificial sweeteners induce glucose intolerance by altering the gut microbiota. Nature 514, 181–186. 25231862 10.1038/nature13793

[11] Suez J , Cohen Y , Valdés-Mas R et al. (2022) Personalized microbiome-driven effects of non-nutritive sweeteners on human glucose tolerance. Cell 185, 3307–3328.e19. 35987213 10.1016/j.cell.2022.07.016

[12] Sylvetsky AC , Kuttamperoor JT , Langevin B et al. (2024) Intergenerational transmission of sucralose and acesulfame-potassium from mothers to their infants via human milk: a pharmacokinetic study. Am J Clin Nutr 120, 846–853. 39111550 10.1016/j.ajcnut.2024.08.001 PMC11473674

[13] Dunford EK , Miles DR , Ng SW et al. (2020) Types and amounts of nonnutritive sweeteners purchased by US households: a comparison of 2002 and 2018 Nielsen Homescan Purchases. J Acad Nutr Diet 120, 1662–1671.e10. 32739278 10.1016/j.jand.2020.04.022 PMC7529721

[14] Hafner E & Pravst I (2021) The sharp rise in the use of low- and no-calorie sweeteners in non-alcoholic beverages in Slovenia: an update based on 2020 data. Front Nutr 8, 778178. 34869543 10.3389/fnut.2021.778178 PMC8640248

[15] Bayram HM & Ozturkcan A (2022) Added sugars and non-nutritive sweeteners in the food supply: are they a threat for consumers? Clin Nutr ESPEN 49, 442–448. 35623850 10.1016/j.clnesp.2022.03.006

[16] Russell C , Baker P , Grimes C et al. (2023) Global trends in added sugars and non-nutritive sweetener use in the packaged food supply: drivers and implications for public health. Public Health Nutr 26, 952–964. 35899782 10.1017/S1368980022001598 PMC10346066

[17] Dunford EK , Taillie LS , Miles DR et al. (2018) Non-nutritive sweeteners in the packaged food supply—an assessment across 4 countries. Nutrients 10, 257. 29495259 10.3390/nu10020257 PMC5852833

[18] Zancheta Ricardo C , Corvalán C , Smith Taillie L et al. (2021) Changes in the use of non-nutritive sweeteners in the Chilean food and beverage supply after the implementation of the food labeling and advertising law. Front Nutr 8, 773450. 34859036 10.3389/fnut.2021.773450 PMC8630583

[19] Secretaria de economia (2010) Amendment to the Mexican Official Standard NOM-051-SCFI/SSA1–2010, General labeling specifications for prepackaged foods and non-alcoholic beverages-Commercial and health information, published on April 5, 2010. https://www.dof.gob.mx/2020/SEECO/NOM_051.pdf (accessed 27 March 2022).

[20] Ministerio de Salud (2022) Decreto 151/2022, Promotion of healthy eating. Buenos Aires, 2022. https://www.argentina.gob.ar/normativa/nacional/decreto-151-2022-362577/texto (accessed 04 November 2022).

[21] Ministerio de Salud y Protección Social (2022). Resolution number 2429 of 2022. https://www.globalfoodresearchprogram.org/wp-content/uploads/2022/12/RESOLUCION-2492-DEL-13-DE-DICIEMBRE-DE-2022-182.pdf (accessed 04 November 2022).

[22] Sylvetsky AC , Greenberg M , Zhao X et al. (2014) What parents think about giving nonnutritive sweeteners to their children: a pilot study. Int J Pediatr 2014, 1–5. 10.1155/2014/819872 PMC4236964 25435883

[23] Grilo MF , Smith Taillie L , Zancheta Ricardo C et al. (2022) Prevalence of low-calorie sweeteners and related front-of-package claims in the Brazilian Packaged Food Supply. J Acad Nutr Diet 122, 1296–1304. 34954081 10.1016/j.jand.2021.12.009 PMC9213564

[24] Beltrá M , Tomás H , López JC et al. (2022) Nutritional description of foods with low-and no-calorie sweeteners in Spain: the BADALI project. Nutrients 14, 2686. 35807866 10.3390/nu14132686 PMC9268128

[25] Ministério da Saúde & Agência Nacional de Vigilância Sanitária (2020) Resolução da Diretoria Colegiada – RDC nº 429, de 8 de outubro de 2020. Provides for the nutrition labeling of packaged foods. https://antigo.anvisa.gov.br/documents/10181/3882585/RDC_429_2020_.pdf/9dc15f3a-db4c-4d3f-90d8-ef4b80537380 (accessed November 2024).

[26] Ministério da Saúde & Agência Nacional de Vigilância Sanitária (2020) Instrução Normativa - IN nº 75, de 8 de outubro de 2020. Establishes the technical requirements for the declaration of nutrition labeling on packaged foods. https://antigo.anvisa.gov.br/documents/10181/3882585/IN+75_2020_.pdf/7d74fe2d-e187-4136-9fa2-36a8dcfc0f8f (accessed November 2024).

[27] Duran AC , Ricardo CZ , Mais LA et al. (2021) Role of different nutrient profiling models in identifying targeted foods for front-of-package food labelling in Brazil. Public Health Nutr 24, 1514–1525. 32515717 10.1017/S1368980019005056 PMC8025091

[28] Figueiredo LDS , Scapin T , Fernandes AC et al. (2018) Where are the low-calorie sweeteners? An analysis of the presence and types of low-calorie sweeteners in packaged foods sold in Brazil from food labelling. Public Health Nutr 21, 447–453. 29072154 10.1017/S136898001700283X PMC10260891

[29] Instituto Brasileiro de Geografia e Estatística (2022) Panorama Belo Horizonte. https://cidades.ibge.gov.br/brasil/mg/belo-horizonte/panorama%0A%0A (accessed November 2024).

[30] Associação Brasileira de Supermercados (2020) Ranking ABRAS 2020. https://www.abras.com.br/edicoes-anteriores/Main.php?MagNo=259 (accessed 11 January 2021).

[31] Centre for Genomic Pathogen Surveillance (2024) Epicollect5. https://five.epicollect.net/ (accessed November 2024).

[32] Tomaz LA , Pereira CG , Braga LV et al. (2022) From the most to the least flexible nutritional profile: classification of foods marketed in Brazil according to the Mexican and Brazilian models. Front Nutr 9, 919582. 36204372 10.3389/fnut.2022.919582 PMC9531871

[33] Ministério da Saúde & Agência Nacional de Vigilância Sanitária (2002) Resolução da Diretoria Colegiada – RDC no 259, de 20 de setembro de 2002 - Approves the Technical Regulation on the Labeling of Packaged Foods. Diário Oficial da União. 23 set 2002. https://bvsms.saude.gov.br/bvs/saudelegis/anvisa/2002/rdc0259_20_09_2002.html (accessed November 2021).

[34] Ministério da Saúde (1998) Portaria SVS/MS nº 29, de 29 de janeiro de 1998. Technical Regulation concerning Foods for Special Dietary Uses. *Diário Oficial da União*, Brasília, 1998. https://bvsms.saude.gov.br/bvs/saudelegis/svs1/1998/prt0029_13_01_1998_rep.html (accessed November 2021).

[35] Ministério da Saúde (1998) Portaria SVS/MS nº 30, de 13 de janeiro de 1998. Technical Regulation concerning Foods for Weight Control. *Diário Oficial da União*, Brasília, 1998. https://www.saude.rj.gov.br/comum/code/MostrarArquivo.php?C=MjIxMA%2C%2C (accessed November 2021).

[36] Ministério da Saúde & Agência Nacional de Vigilância Sanitária (2012) Resolução RDC nº 54, de 12 de novembro de 2012. Provides for the Technical Regulation on Complementary Nutrition Information. *Diário Oficial da União*, Brasília, 2012. https://bvsms.saude.gov.br/bvs/saudelegis/anvisa/2012/rdc0054_12_11_2012.html (accessed November 2021).

[37] Presidência da República (2009) Decreto nº 6.871, de 04 de junho de 2009. Regulates Law No. 8,918 of July 14, 1994, which provides for the standardization, classification, registration, inspection, production, and oversight of beverages. *Diário Oficial da União*, Brasília, 2009. https://www.planalto.gov.br/ccivil_03/_ato2007-2010/2009/decreto/d6871.htm (accessed November 2021).

[38] Coyle DH , Dunford EK , Wu JH et al. (2021) The use of non-nutritive and low-calorie sweeteners in 19 915 local and imported pre-packaged foods in Hong Kong. Nutrients 13, 1861. 34072564 10.3390/nu13061861 PMC8229473

[39] Mora-Plazas M , Gómez LF , Miles DR et al. (2019) Nutrition quality of packaged foods in Bogotá, Colombia: a comparison of two nutrient profile models. Nutrients 11, 1011. 31060219 10.3390/nu11051011 PMC6567873

[40] Carvalho TEM , Waisenberg A , Sato PD et al. (2022) Consumer perceptions of non-caloric sweeteners and the content of caloric and non-caloric sweeteners in ultra-processed products in Brazil. Cienc e Saude Coletiva 27, 1989–2000. 10.1590/1413-81232022275.08452021 35544825

[41] Dunford EK , Coyle DH , Louie JCY et al. (2022) Changes in the presence of nonnutritive sweeteners, sugar alcohols, and free sugars in Australian Foods. J Acad Nutr Diet 122, 991–999.e7. 34864247 10.1016/j.jand.2021.11.018

[42] Goodman S , Vanderlee L , Jones A et al. (2021) Perceived healthiness of sweeteners among young adults in Canada. Can J Dietetic Pract Res 82, 90–94. 10.3148/cjdpr-2020-030 33320777

[43] Takehara CT , Nicoluci ÍG , Andrade TFS et al. (2022) A comprehensive database of declared high-intensity sweeteners in Brazilian commercial products and updated exposure assessment. Food Res Int 161, 111899. 36192918 10.1016/j.foodres.2022.111899

[44] de Diniz JA , Pedreira ME , Moore SR et al. (2023) Artificial sweeteners: regulation in Brazil, technological implications in food production and health. Revista Uningá 59, eUJ4280.

[45] Ul-Ain Q , Sikander M , Khan SA et al. (2016) Low calorie intense sweeteners safety aspects. In Sweeteners. Reference Series in Phytochemistry, pp. 591–612 [ JM Merillon & K Ramawat , editors]. Cham: Springer.

[46] Ministério da Saúde & Agência Nacional de Vigilância Sanitária (2019) Resolução da Diretoria Colegiada - RDC nº 281, de 29 de abril de 2019. Authorizes the use of food additives and processing aids in various categories of foods. Diário Oficial da União, Brasília, 2019. https://bvsms.saude.gov.br/bvs/saudelegis/anvisa/2019/rdc0281_29_04_2019.pdf (accessed November 2021).

[47] Grembecka M (2015) Sugar alcohols—their role in the modern world of sweeteners: a review. Eur Food Res Technol 241, 1–14.

[48] Agência Nacional de Vigilância Sanitária (2023) Panel on food additives.. https://app.powerbi.com/view?r=eyJrIjoiZmQ2ZDBjNTItMDFmMi00MmM5LWE4Y2QtMzBhOGZlYTU4OGUzIiwidCI6ImI2N2mMjNmLWMzZjMtNGQzNS04MGM3LWI3MDg1ZjVlZGQ4MSJ9&pageName=ReportSection08a3239a66872bb5b7a9 (accessed 04 November 2022).

[49] de Sousa RCS , de Fatima Gomides M , Costa K et al. (2023) Optimization and validation of an analytical method for the determination of sweeteners in beverages by HPLC-ELSD. Food Anal Methods 17, 207–225.

[50] Codex Alimentarius (2015) General Standard for Food Additives Codex Stan 192–1995. https://www.fao.org/fao-who-codexalimentarius/shproxy/en/?lnk=1&url=https%253A%252F%252Fworkspace.fao.org%252Fsites%252Fcodex%252FStandards%252FCXS%2B1921995%252FCXS_192e.pdf (accessed 02 November 2022).

[51] Ministério da Saúde & Agência Nacional de Vigilância Sanitária (2021) Resolução RDC no 588, de 20 de dezembro de 2021. Authorizes the use of food additives and processing aids in various categories of foods. *Diário Oficial da União*, Brasília, 2021. https://antigo.anvisa.gov.br/documents/10181/6253875/rdc_588_2021_.pdf/683e12f8-baf1-4969-b479-ab031a30db9b (accessed November 2021).

[52] Zygler A , Wasik A & Namieśnik J (2009) Analytical methodologies for determination of artificial sweeteners in foodstuffs. TrAC, Trends Anal Chem 28, 1082–1102.

[53] Giménez A , de Saldamando L , Curutchet MR et al. (2017) Package design and nutritional profile of foods targeted at children in supermarkets in Montevideo, Uruguay. Cad Saude Publica 33, e00032116. 28614447 10.1590/0102-311X00032116

[54] Stoltze FM , Barker JO , Kanter R et al. (2018) Prevalence of child-directed and general audience marketing strategies on the front of beverage packaging: the case of Chile. Public Health Nutr 21, 454–464. 29094661 10.1017/S1368980017002671 PMC10260837

[55] Amanda Rutiquewiski Gomes (2020) A influência dos apelos visuais na intenção de consumo de cereais matinais destinados ao público infantil: um estudo da linguagem gráfica nas embalagens. Dissertação (mestrado), Universidade Federal do Paraná.

[56] Roncarelli S & Ellicott C (2010) Packaging Essentials: 100 Design Principles for Creating Packages. Beverly, MA: Rockport Publishers. p. 208.

[57] Sylvetsky AC , Walter PJ , Garraffo HM et al. (2017) Widespread sucralose exposure in a randomized clinical trial in healthy young adults. Am J Clin Nutr 105, 820–823. 28228424 10.3945/ajcn.116.144402 PMC5366047

[58] Secretaría de salud (2012) Agreement establishing the additives and processing aids in foods, beverages and dietary supplements, their use, and health provisions (Continued in the Fourth Section). Ciudad de México, 2012. https://dof.gob.mx/nota_detalle_popup.php?codigo=5259470 (accessed 02 November 2022).

[59] Ministerio de salud (1996) Sanitary Food Regulation dto. N° 977/96 (d.of. 13.05.97). Santiago, 1996. https://www.minsal.cl/sites/default/files/files/DECRETO_977_96%20actualizado%20a%20Enero%202015(1).pdf (accessed 02 November 2022).

